# RNA-Guided Cas9-Induced Mutagenesis in Tobacco Followed by Efficient Genetic Fixation in Doubled Haploid Plants

**DOI:** 10.3389/fpls.2016.01995

**Published:** 2017-01-04

**Authors:** Sindy Schedel, Stefanie Pencs, Götz Hensel, Andrea Müller, Twan Rutten, Jochen Kumlehn

**Affiliations:** ^1^Plant Reproductive Biology, Physiology and Cell Biology, Leibniz Institute of Plant Genetics and Crop Plant Research (IPK)Gatersleben, Germany; ^2^Structural Cell Biology, Physiology and Cell Biology, Leibniz Institute of Plant Genetics and Crop Plant Research (IPK)Gatersleben, Germany

**Keywords:** genome engineering, haploid technology, pollen embryogenesis, site-directed mutagenesis, mutant fixation

## Abstract

Customizable endonucleases are providing an effective tool for genome engineering. The resulting primary transgenic individuals (T_0_) are typically heterozygous and/or chimeric with respect to any mutations induced. To generate genetically fixed mutants, they are conventionally allowed to self-pollinate, a procedure which segregates individuals into mutant heterozygotes/homozygotes and wild types. The chances of recovering homozygous mutants among the progeny depend not only on meiotic segregation but also on the frequency of mutated germline cells in the chimeric mother plant. In *Nicotiana* species, the heritability of Cas9-induced mutations has not been demonstrated yet. RNA-guided Cas9 endonuclease-mediated mutagenesis was targeted to the *green fluorescent protein* (*GFP*) gene harbored by a transgenic tobacco line. Upon retransformation using a *GFP*-specific guide RNA/Cas9 construct, the T_0_ plants were allowed to either self-pollinate, or were propagated via regeneration from *in vitro* cultured embryogenic pollen which give rise to haploid/doubled haploid plants or from leaf explants that form plants vegetatively. Single or multiple mutations were detected in 80% of the T_0_ plants. About half of these mutations proved heritable via selfing. Regeneration from *in vitro* cultured embryogenic pollen allowed for homozygous mutants to be produced more efficiently than via sexual reproduction. Consequently, embryogenic pollen culture provides a convenient method to rapidly generate a variety of genetically fixed mutants following site-directed mutagenesis. The recovery of a mutation not found among sexually produced and analyzed progeny was shown to be achievable through vegetative plant propagation *in vitro*, which eventually resulted in heritability when the somatic clones were selfed. In addition, some in-frame mutations were associated with functional attenuation of the target gene rather than its full knock-out. The generation of mutants with compromised rather than abolished gene functionality holds promise for future approaches to the conclusive functional validation of genes which are indispensible for the plant.

## Introduction

Site-specific modifications to genomic DNA sequence, induced by either customizable zinc finger nucleases (ZFNs, Kim et al., 1996), transcription activator-like effector nucleases (TALENs, Christian et al., 2010) or the more recently established RNA- or DNA-guided endonucleases (RGENs, Jinek et al., 2012; Shan et al., 2013; Zetsche et al., 2015; NgAgo, Gao et al., 2016), represents the new frontier in genetic engineering. All of these platforms involve a customizable DNA-binding module (proteinaceous in the case of ZFN and TALEN, and complementary nucleic acid molecules in the case of RGEN and NgAgo) along with a generic enzymatic DNA-cleavage module (FokI for ZFNs and TALENs, Cas9 or Cpf1 for RGENs and Argonaute in case of the NgAgo platform). The endonuclease’s function is to generate a double strand break (DSB) in, or close to the target site, which is then repaired by either error-prone non-homologous end-joining (NHEJ) or the more precise homology-directed repair (HDR) pathway (Waterworth et al., 2011). Whereas NHEJ tends to induce random insertions, deletions, or substitutions at the target DSB site, HDR can be combined with a synthetic repair template to generate predictable sequence modifications.

Effective guide RNA (gRNA)/Cas9-mediated heritable mutagenesis has been demonstrated in both di- and monocotyledonous plant species such as Arabidopsis (Feng et al., 2014; Jiang et al., 2014; Hyun et al., 2015; Ma et al., 2015; Eid et al., 2016), rice (Zhang et al., 2014; Zhou et al., 2014; Ma et al., 2015; Xu et al., 2015), tomato (Brooks et al., 2014; Pan et al., 2016), rape seed and barley (Lawrenson et al., 2015). Whereas heritable NHEJ-mediated mutation has been shown in a range of species (for review see Ma et al., 2016), so far the use of HDR has been confined to just a few situations (Schiml et al., 2014; Čermák et al., 2015; Li et al., 2015; Svitashev et al., 2015; Zhao et al., 2016). The heritability of induced mutations was examined in several plant species with the consistent result that not all of the induced mutations found in a given population of primary mutants were re-detected among the analyzed progeny. For example, in a study focusing on *Arabidopsis thaliana*, about 76% of mutations uncovered in primary mutant plants were detected also in the next generation (Feng et al., 2014). In this context, it is worth to mention that the identification of mutations is dependent upon the tissue sampling representation and frequency as well as the sensitivity of the molecular detection method. Depending on the plant species and the transformation method, mutant alleles can be heterozygous or homozygous. Even though non-chimeric individuals can instantly occur, all primary mutants should be considered putatively chimeric until proven otherwise. Consequently, it is necessary to separate and genetically fix mutations by screening the T_1_ generation, a process which is relatively labor-intensive and time-consuming. Selections are then validated in the T_2_ generation. gRNA/Cas9-mediated mutagenesis in the genus *Nicotiana* was first demonstrated at the cellular level in *Nicotiana benthamiana* (Li et al., 2013; Nekrasov et al., 2013), since when its effectiveness has also been established in *Nicotiana tabacum* plants (Gao et al., 2014). However, the inheritance of those induced mutations has not been demonstrated.

The present comprehensive study details a number of heritable gRNA/Cas9-induced mutations to the *GFP* transgene used here as experimental target in tobacco. It aims to demonstrate that induced mutations are heritable and that genetic fixation of altered sequences can be particularly efficiently achieved by regeneration of progeny plants from *in vitro* cultured embryogenic pollen. In addition, an attempt was made to use vegetative propagation for the maintenance of mutations which were induced in primary transgenic plants.

## Materials and Methods

### Plant Growth

Seeds of the wild type (WT) *N. tabacum* accession SR1 and the single *GFP* transgene copy line TSP20L1-1 were surface-sterilized and germinated for 2 weeks on solidified Murashige and Skoog (MS) medium (Murashige and Skoog, 1962). They were subsequently transferred into boxes (107 × 94 × 96 cm) containing MS medium and left to grow for 6–8 weeks.

Once regenerants from tissue culture (see below) had developed a viable root system, they were potted into soil and grown under a 16 h photoperiod provided by 35,000 lux light at 22/20°C, then re-potted and grown for a further ∼10 weeks (20/18°C, 16 h photoperiod, 30,000 lux) to obtain progeny by self-fertilization. T_1_ seed was germinated in soil under 22/20°C day/night, 16 h photoperiod (35,000 lux) light.

### T-DNA Constructs

The *GFP* sequence was amplified using the primer pair GH-SpeI-GFP F1/GH-NcoI-GFP R1 (Supplementary Table S1) and introduced into the pCR2.1 plasmid (Invitrogen, Carlsbad, CA, USA) to form pGH124. A *GFP-*containing *Spe*I/*Eco*RI fragment of pGH124 was inserted into pNos-AB-M (DNA Cloning Service, Hamburg, Germany) to yield pGH119. Subsequently, the *Spe*I/*Hin*dIII *GFP*-containing fragment was subcloned into pUbiAT-OCS (DNA Cloning Service) between the *A. thaliana UBIQUITIN-10* promoter and the *Agrobacterium tumefaciens OCS* termination sequence, the resultant plasmid being denoted pGH167. A *Sfi*I fragment of pGH167 harboring the entire *GFP* expression cassette was integrated into the binary vector pLH9000 (DNA-Cloning-Service) to produce pGH292, which was introduced into *A. tumefaciens* strain GV2260 via a heat shock protocol.

The Gateway^®^-compatible RNA-guided Cas9 expression system (Fauser et al., 2014) was used to construct a *GFP*-specific derivative. In the first step, the *GFP*-specific protospacer sequence (annealed oligonucleotides, Supplementary Table S1) was introduced into pEN-Chimera by exploiting its two *Bbs*I sites. The resulting gRNA-encoding chimera, driven by the *A. thaliana U6-26* promoter, was then transferred into pDe-CAS9 through a single site Gateway^®^ LR reaction to form an expression cassette where the *Cas9* (codon optimized for *A. thaliana*) was driven by the *Petroselinum crispum UBIQUITIN-4-2* promoter and its terminator sequence was *pea3A* from *Pisum sativum*. The resulting binary vector (pSI24) was introduced into *Agrobacterium* as described above.

### Tobacco Transformation

Fully developed leaves of 6–8 week old plants were cut into ∼1 cm^2^ pieces and cultured on MS medium containing 3% (w/v) sucrose, 1 mg/L 6-benzylaminopurine, 0.1 mg/L 1-naphthalene acetic acid, and 0.8% (w/v) bacto agar. After 1 or 2 days, the leaf segments were bathed in a suspension of *Agrobacterium* cells (*OD*_600_ = 0.2) for 30 min, blotted dry and then replaced on the culture medium and held in the dark at 19°C for 3 days. The leaf segments were subsequently removed to the same medium containing 400 mg/L Ticarcillin and either 100 mg/L kanamycin in the case of transformation with strain GV2260/pGH292 or 5 mg/L Bialaphos when strain GV2260/pSI24 was used, and sub-cultured every 10 days (initially in the dark, then in the light) at 22°C. Differentiated shoots were separated from the callus and transplanted into a root induction MS-based medium containing 2% (w/v) sucrose, 0.8% (w/v) bacto agar and antibiotics (as described above). Rooted plantlets were later transferred to soil and grown in a greenhouse to maturity.

### Vegetative *In vitro* Propagation from Leaf Explants

In order to vegetatively propagate the T_0_ plant #125, explants from different leaves were surface-sterilized by immersion first in 70% (v/v) ethanol and then in 0.6% (v/v) sodium hypochlorite, then rinsed three times in sterile water. A number of ∼1 cm^2^ pieces was cut and placed on MS medium containing 3% (w/v) sucrose, 1 mg/L 6-benzylaminopurine, 0.1 mg/L 1-naphthalene acetic acid, 0.8% (w/v) bacto agar, 5 mg/L Bialaphos, and 400 mg/L Ticarcillin. Regeneration was handled as for the transgenic material, as described above.

### Embryogenic Pollen Culture

The procedure used for embryogenic pollen culture followed Floss et al. (2009). In short, closed flower buds were sterilized; the anthers squeezed out and transferred to a Waring Blender for the homogenization of the tissue and the release of immature pollen grains. After filtration of the homogenate the pollen grains were washed several times with medium and the numbers were counted using a haemocytometer. The pollen grain suspension with ∼500,000 pollen grains/mL was first incubated at 32°C for 1 week and after medium change for another 3 weeks at 25°C. The developed embryo-like structures were spread on solid medium to enable shoot and root formation and rooted plantlets were then transferred to the greenhouse. The selection of regenerants was based on the presence in the medium of 5 mg/L Bialaphos.

### Genotyping

Genomic DNA was isolated from two leaf disks of 11 mm diameter taken from one leaf of 4–5 weeks old plants using a phenol chloroform-based procedure (Pallotta et al., 2000) to serve as a template for PCRs targeting *Bar, Cas9*, and gRNA, employing primer pairs as listed in Supplementary Table S1. To detect mutations in *GFP*, the genomic region surrounding the target sequence was PCR amplified using the primer pair GH-GFP R2/F1 (Supplementary Table S1), and the PCR product, following its purification using a QIAquick PCR purification kit (QIAGEN, Hilden, Germany), was subjected to the T7E1 assay (NEB, Ipswich, MA, USA). Toward this end, the PCR product was denatured and re-annealed according to the manufacturer’s protocol and treated with 5 U of T7E1 for 45 min at 37°C. The analysis was done using 1.5% agarose gel electrophoresis. PCR products scored as T7E1-positive were cloned into the pGEM^®^-T Easy vector (Promega, Madison, WI, USA) following the manufacturer’s protocol and resultant clones were individually sequenced.

For the analysis of progeny plants, the PCR products of those samples flagged as T7E1-negative (indicating the presence of no more than one *GFP* variant) were sequenced directly in order to discriminate between *GFP* WT and mutant homozygotes. Since the T7E1 assay detects induced mutations only at frequencies of ∼1% or greater (Voytas, 2013) it is possible to get false-negative results, but these were then uncovered by direct sequencing. Several T7E1-positive progeny (i.e., those harboring two or more *GFP* variants) were examined in more detail by generating and sequencing individual clones from their *GFP*-specific PCR product (see previous paragraph). The portion of *GFP* WT plants, homozygous mutants, and heterozygous/chimeric plants was calculated according to the results of the T7E1 assay and the direct sequencing of PCR product.

### Confocal Microscopy

Green fluorescent protein fluorescence in leaves of selected homozygous *GFP* mutants and WT plants was detected by using the Zeiss LSM780 confocal laser scanning microscope (Carl Zeiss, Jena, Germany). Visualization of the GFP signal was performed using a 488 nm laser line in combination with a 491–530 nm bandpass. To improve the differentiation of fluorescence signals, all pictures were identically edited using Adobe Photoshop CS (Adobe Systems, San José, CA, USA) as follows: tonal correction from 255 to 100, the blue and magenta tones were increased to +25 each, and the cyan tones decreased to -5. The chroma and lab-brightness of the blue tones was decreased to -50.

## Results

**FIGURE 1:**
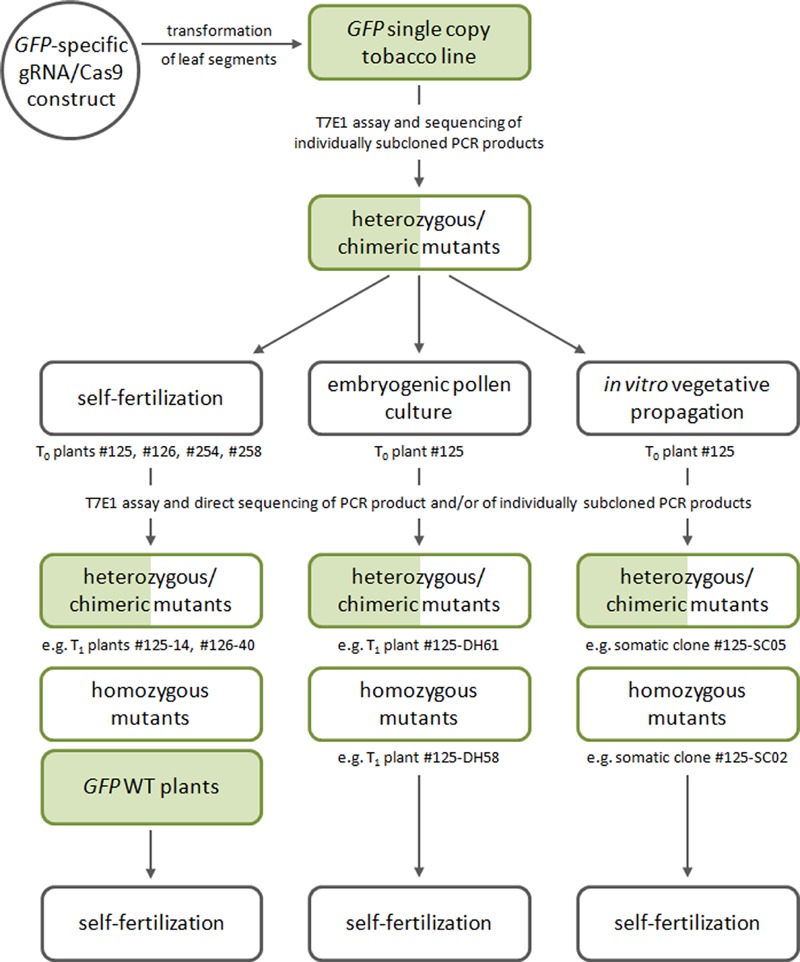
**General workflow of the study involving the generation of gRNA/*Cas9* primary transgenic/*GFP* mutant plants, which is followed by various approaches to the generation of mutant progeny (via selfing, production of doubled haploids, or vegetative propagation)**.

The workflow of the study is summarized in **Figure 1**, which includes the generation of gRNA/*Cas9* primary transgenic/*GFP* mutant plants as well as the various approaches pursued to obtain progeny segregating with regards to the transmitted mutant alleles.

### gRNA/Cas9-Mediated Genetic Modification of *GFP*

**FIGURE 2:**
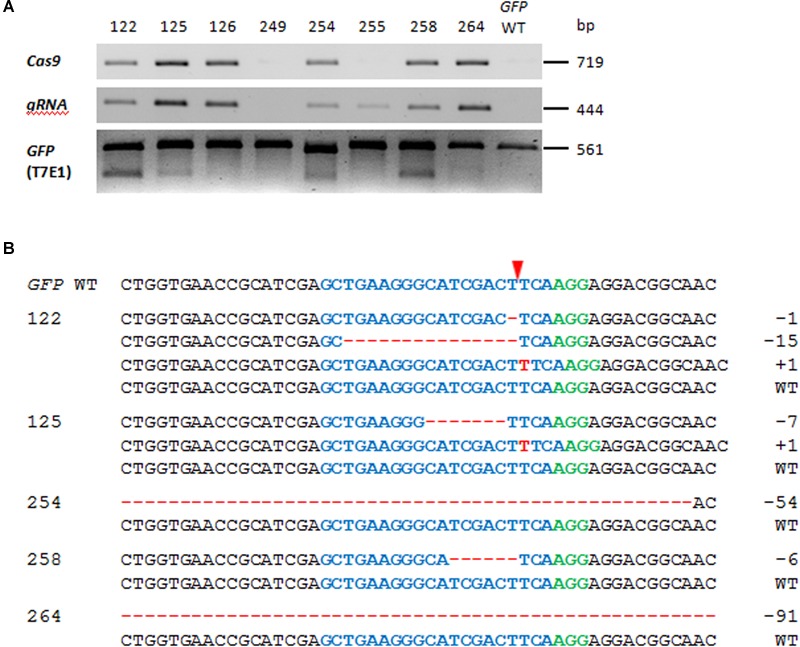
**Modifications of the *green fluorescent protein (GFP)* sequence induced by RNA-guided Cas9 endonuclease. (A)** The presence of *Cas9* and gRNA and induced mutations to *GFP* in the T_0_ generation. The first two rows show the outcomes of PCRs targeting *Cas9* and gRNA in representative T_0_ plants and the wild type *GFP* (*GFP* WT). The lower row shows the outcome of the T7E1 assay. The two cleavage products were of similar length and thus resolved electrophoretically only as a single band. **(B)** Sequencing outcomes of individually cloned *GFP* sequences from five T_0_ plants. The numbers of induced nucleotide changes are indicated to the right of each sequence. The sequence colored blue is the protospacer and the one colored green is the protospacer-adjacent motif (PAM); the red arrowhead indicates the cleavage site. Deletions are represented by red dashes and insertions by red letters.

A T-DNA construct comprising a *GFP*-specific gRNA, *Cas9*, and *Bar* (encoding phosphinothricin acetyltransferase, the activity of which ensures plant resistance to the herbicide Bialaphos) was introduced into a transgenic tobacco line harboring a single copy of *GFP*, along with *NptII* (encoding neomycin phosphotransferase for plant resistance to kanamycin). When the presence of the *Cas9* and the gRNA sequences in the genomic DNA extracted from Bialaphos-resistant regenerants was tested by PCR, 15 of the 21 regenerants were shown to harbor both transgene sequences. The *GFP* region in these 15 individuals was then characterized using the T7 endonuclease (T7E1) assay. The 561 bp *GFP*-specific PCR products were melted and re-annealed, in order to produce heteroduplex DNA when more than one variant of the sequence was present; this structure is cleavable by T7E1 (Mashal et al., 1995). The T7E1 profile of the PCR products derived from 12 of the 15 gRNA/*Cas9*-positive plants comprised two fragments of similar size, which resolved as a single band following 1.5% agarose gel electrophoresis (**Figure 2A**). By contrast, none of the six transgenic plants lacking *Cas9* and/or gRNA scored positive with respect to the T7E1 assay. The sequencing of individual clones derived from the *GFP*-specific PCR products of five of the 12 mutation-carrying plants (#122, #125, #254, #258, and #264) revealed the presence of deletions (ranging in length from 1 to 91 bp) or insertions of 1 bp (**Figure 2B**). Across all T_0_ plants examined, 62.5% of the independently generated mutations caused a translational reading frame shift, and 60% of these frame shifts were associated with the formation of premature stop codons. Some of the deletions (corresponding to a loss of 2–18 amino acids in the gene product) did not induce a reading frame shift. Plants #122 and #125 both harbored more than one mutated *GFP* sequence, while the other three plants only harbored a single one. In all five T_0_ mutants examined in detail, the WT *GFP* sequence was retained, indicating that the plants were either heterozygous and/or chimeric with respect to the induced mutations.

### Sexual Transmission of the *GFP* Mutations

**FIGURE 3:**
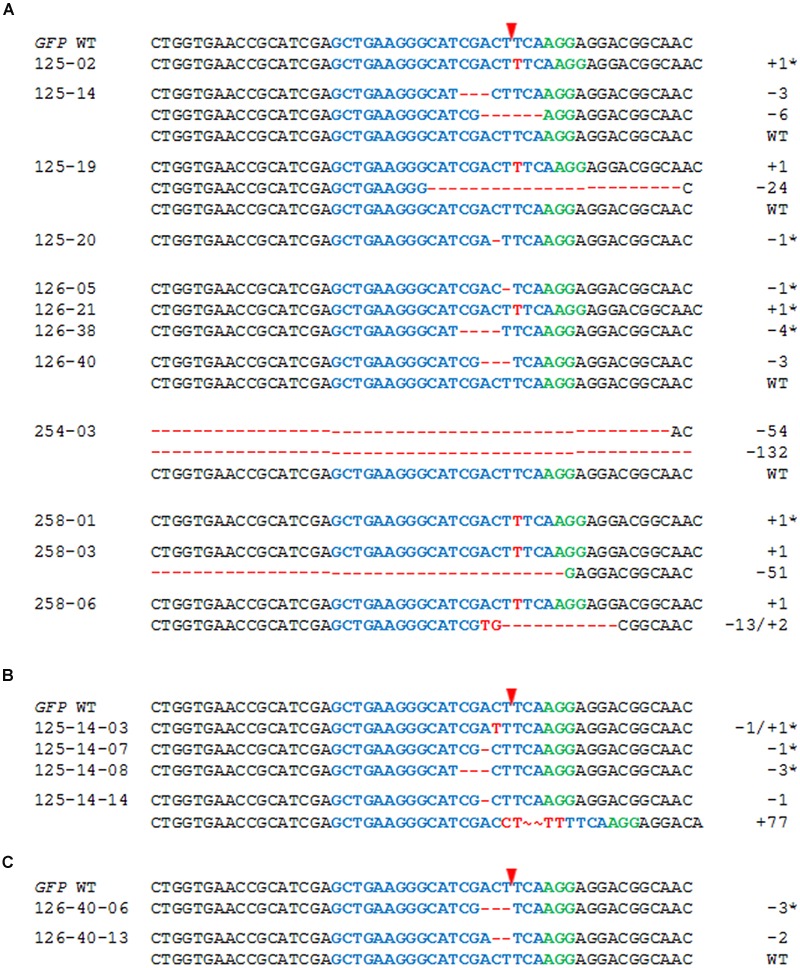
**The inheritance of targeted mutagenesis products. (A)** Alignment of the *GFP* sequences of T_1_ progeny of T_0_ plants #125, #126, #254, and #258 and of the descendants of heterozygous/chimeric T_1_ plants **(B)** #125-14, and **(C)** #126-40. The numbers of nucleotides deleted (red dashes) and inserted (red letters) are shown to the right of each sequence. The sequence colored blue is the protospacer sequence and the one colored green is the PAM; the red arrowhead indicates the cleavage site. Homozygous mutant plants are indicated by asterisks.

T_1_ progeny of plants #125, #254, and #258 (each carrying the WT and mutant *GFP* sequences), and #126 (harboring no detectable *GFP* mutation despite the presence of gRNA and *Cas9*) was analyzed in detail. Between 15 and 50 progeny per selfed T_0_ plant were subjected to the T7E1 assay and further genotyping by sequencing (see “Materials and Methods”), to discriminate between heterozygous/chimeric mutant plants, WT and mutant homozygotes. The T_1_ progenies were shown to include some non-WT individuals in each case (**Figure 3A**; **Table 1**). Among the 25 T_1_ offspring of plant #125 analyzed in detail, the 1 bp insertion segregated, but the 7 bp deletion was not identified among the progeny generated via selfing. Four deletions (1, 3, 6, and 24 bp) were uncovered in the T_1_ generation even though these had not been detected in the mother plant. In plant #254, a 54 bp deletion proved to be heritable, and a 132 bp deletion was additionally revealed in the T_1_ generation. In contrast, a 6 bp deletion mutation of plant #258 was not identified among the seven T_1_ plants which were analyzed in detail, but three new mutations were detected. In all, half of the *GFP* mutations detected in T_0_ plants #125, #254, and #258 were re-detected among the analyzed descendants, while the majority (66.7%) of all independently generated mutations detected in T_0_ and/or T_1_ plants was found in the T_1_ generation for the first time (**Table 1**). Mutations were also uncovered among the progeny of plant #126: specifically, three micro-deletions (1, 3, and 4 bp) and the identical 1 bp insertion that was independently induced in the T_0_ plant #125.

**Table 1:** The frequency of mutations among progeny of the T_0_ mother plants produced by either self-fertilization or passage through embryogenic pollen culture.

	Selfing					Haploid technology
T_0_ plant identifier	#125	#126	#254	#258	Average	#125
No. of T_1_ plants analyzed	50	50	15	15	32.5	62
Non-mutant T_1_ plants	5 (10%)	11 (22%)	4 (26.7%)	1 (6.7%)	5.3 (16.3%)	0
Mutant T_1_ plants	45 (90%)	39 (78%)	11 (73.3%)	14 (93.3%)	27.2 (83.7%)	62 (100%^∗^)
Heterozygous/chimeric T_1_ mutants	28 (56%)	29 (58%)	11 (73.3%)	10 (66.6%)	19.5 (63.5%)	25 (40.3%)
Genetically fixed T_1_ mutants	17 (34%)	10 (20%)	0	4 (26.7%)	7.8 (20.2%)	37 (59.7%)
No. of mutations found in T_0_	2	0	1	1	1.3^†^	2
No. of mutations found in T_1_	5	4	2	3	3.3^†^	12
(a) No. of mutations found in T_0_ and T_1_	1	–	1	0	0.6^†^	2
Not genetically fixed in T_1_ plants	0	–	1	0	0.3^†^	0
Genetically fixed in T_1_ plants	1	–	0	0	0.3^†^	2
(b) No. of mutations not found in T_0_ but found in T_1_	4	4	1	3	2.7^†^	10
Not genetically fixed in T_1_ plants	3	1	1	2	2.0^†^	3
Genetically fixed T_1_ plants	1	3	0	1	0.7^†^	7

*^∗^Pollen embryogenesis was conducted under Bialaphos-based selection for gRNA/*Cas9*-transgenics.*

*^†^Calculation conducted without plant #126, which did not carry any detectable mutation in T_0_.*

In all, 56% of the plant #125 T_1_ progeny carried one or more mutated *GFP* sequences in addition to the WT *GFP* allele, 34% were homozygous for a mutant *GFP* allele and the remaining 10% inherited no *GFP* mutation. Of the plant #254 T_1_ progeny analyzed, 73.4% were heterozygous/chimeric plants and the other 26.6% carried only the WT *GFP* allele, whereas no T_1_ plant was found to be homozygous for a mutation. Among the analyzed T_1_ individuals from plants #126 and #258, the majority were heterozygous/chimeric plants. While homozygous mutants and *GFP* WT plants occurred at a 1-to-1 ratio in the #126 progeny, *GFP* WT individuals were less frequent than mutants in the #258 progeny (**Table 1**). Among all 31 genetically fixed T_1_ mutant individuals derived from plants #125, #126, and #258, six lacked the gRNA/*Cas9*-encoding T-DNA (as shown by a PCR) but carried an identical 1 bp insertion. However, the mutations occurring in the progenies of plants #125, #126, and #258 must have derived from independent events. Of the heterozygous/chimeric T_1_ progeny, 22 also lacked *Cas9*.

In a further investigation, the progeny of selected selfed T_1_ mutant plants was analyzed and two examples are presented in **Figures 3B,C**. Among 14 T_2_ individuals derived from the chimeric plant #125-14, two or more *GFP* sequences were found in five plants, while the remaining ones carried a genetically fixed mutation. Ten T_2_ plants were examined in detail and the maternal 3 bp deletion but not a 6 bp deletion that had first occurred in the T_1_ was detected; new mutations were identified including a 77 bp insertion (**Figure 3B**). In comparison with these results, the analysis of 12 T_2_ individuals of plant #126-40 (heterozygous/chimeric) showed that one individual was homozygous for the *GFP* WT allele, while two plants carried a maternal 3 bp deletion at genetically fixed state and the remaining ones harbored two or more *GFP* variants. Subclones of the *GFP*-specific PCR product of one of these heterozygous/chimeric plants were sequenced whereby a 2 bp deletion was identified beside the *GFP* WT sequence (**Figure 3C**).

### Efficient Production of Non-chimeric, Homozygous Mutants by Means of Pollen Embryogenesis

**FIGURE 4:**
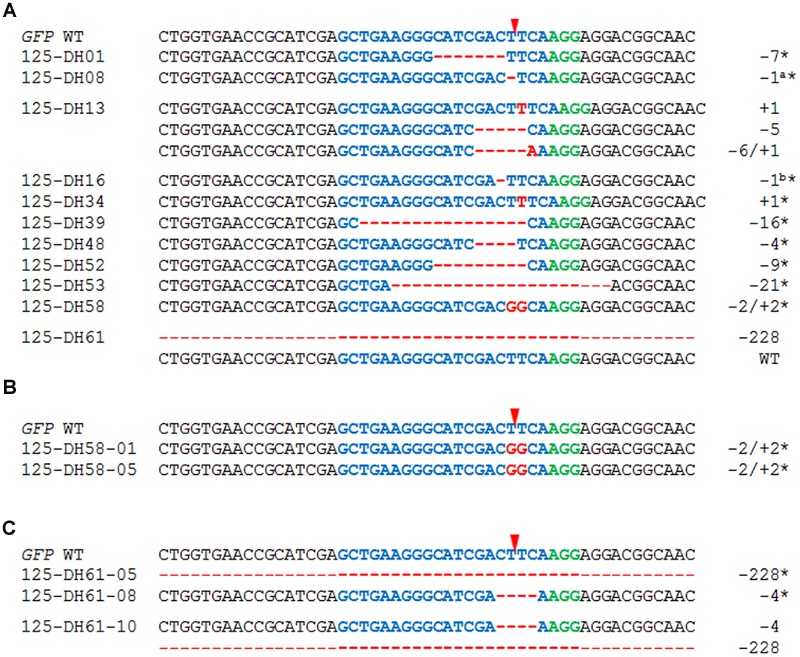
**Separating mutated sequences by regeneration from embryogenic pollen culture. (A)** The sequences of various regenerants derived from T_0_ plant #125 are shown. Alignments of the *GFP* sequences of descendants **(B)** of the homozygous T_1_ plant #125-DH58 and **(C)** of the heterozygous/chimeric T_1_ plant #125-DH61. The numbers of nucleotide changes are indicated to the right of each sequence. The sequence colored blue is the protospacer sequence and the one colored green is the PAM; the red arrowhead indicates the cleavage site. Deletions are represented by red dashes and insertions by red letters; homozygous mutant plants are indicated by asterisks.

In an attempt to accelerate the recovery of non-chimeric, homozygous mutants from chimeric #125 T_0_ mother plant, an embryogenic pollen culture was initiated. All of the 62 Bialaphos-resistant regenerants produced via pollen embryogenesis retained the gRNA/*Cas9*-encoding T-DNA and every plant carried at least one altered *GFP* sequence (**Table 1**) as was revealed by genotyping. In 59.7% of all examined plants produced via pollen embryogenesis, only single mutated *GFP* sequences were detected and the WT *GFP* sequence was absent, suggesting the successful fixation of the mutant sequence. Both of the mutations present in the T_0_ generation (a 1 bp insertion and a 7 bp deletion) were represented among these regenerants, along with 10 additional mutations: these included a 2 bp substitution (plant #125-DH58; **Figure 4A**). In the remaining 40.3% of the regenerants at least two *GFP* variants were present. The sequencing of individual clones of the *GFP*-specific PCR product produced from five of these 25 regenerants revealed that in four, a single altered *GFP* sequence was accompanied by the WT sequence, while in plant #125-DH13 there were only three distinct non-WT sequences (**Figure 4A**); one of the variants involved both a 6 bp deletion and a 1 bp insertion.

To verify the homozygous state of mutant plant #125-DH58, the *GFP*-specific PCR products of five T_2_ individuals were directly sequenced; in all plants, the maternal substitution mutation was recovered in the absence of the *GFP* WT allele (**Figure 4B**). Also 14 offspring plants from the heterozygous/chimeric plant #125-DH61 were analyzed to investigate the transmission of the large 228 bp deletion. The result of the gel electrophoresis of the *GFP*-specific PCR products already showed that in seven plants there were two or more *GFP* variants present. Further five plants showed one band with smaller size than the *GFP* WT control and the direct sequencing of the PCR products of these plants confirmed the 228 bp deletion mutation. The remaining two plants both harbored a 4 bp deletion in the absence of the *GFP* WT allele (**Figure 4C**). In total, 50% of the analyzed T_2_ generation of plant #125-DH61 were heterozygous/chimeric, while the other half carried one mutated *GFP* allele in genetically fixed state. All examined T_2_ plants harbored the gRNA/*Cas9*-encoding T-DNA.

### Maintaining Mutations via *In vitro* Vegetative Propagation

**FIGURE 5:**
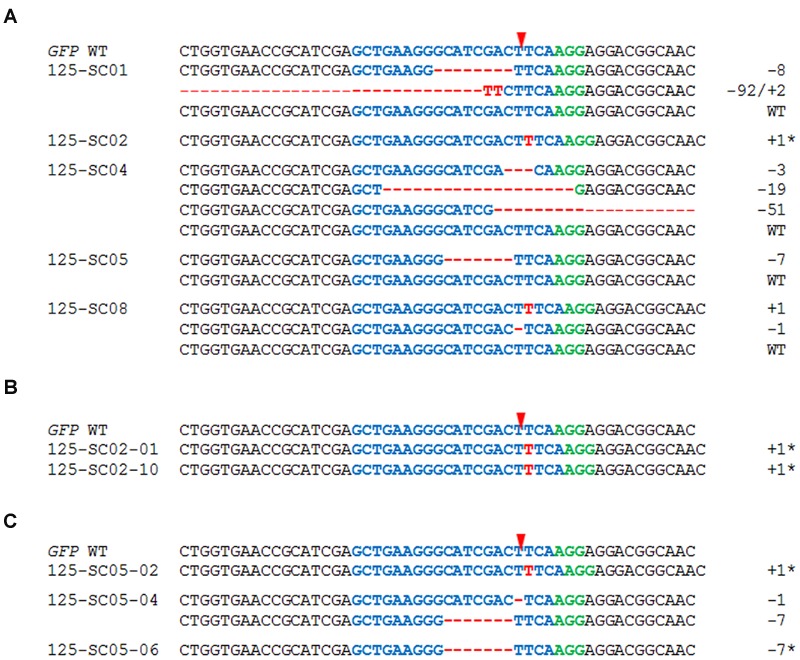
**RNA-guided Cas9 endonuclease-induced mutations in the *GFP* sequence among regenerants derived from leaf explants from chimeric T_0_ plant #125 and their transmission to the next generation. (A)** The figure shows an alignment of the *GFP* sequences recovered as well as the sequences of T_1_ plants **(B)** from the homozygous regenerant #125-SC02 and **(C)** from the heterozygous/chimeric regenerant #125-SC05. The number of nucleotides deleted (red dashes) and inserted (red letters) are shown to the right of each sequence. The sequence colored blue is the protospacer sequence and the one colored green is the PAM; the red arrowhead indicates the cleavage site. Homozygous mutant plants are indicated by asterisks.

The heritability of an induced mutation depends on whether or not the mutated cell belongs to the plant’s germline tissue which gives rise to the gametophytes. Not all of the mutations identified and characterized in the T_0_ plants were detected in the analyzed progeny, as shown above for plant #125 and #258. Thus, an attempt was made to maintain some of these mutations via vegetative propagation. Leaf segments from the T_0_ plant #125 were cultured *in vitro*, from which 15 Bialaphos-resistant regenerants were recovered. A T7E1 assay of their *GFP*-specific PCR products indicated the retention of more than one *GFP* variant in 13 of the 15 plants obtained via vegetative propagation. Detailed genotyping of four of these showed that in one case (plant #125-SC05) there remained only a single altered sequence (the 7 bp deletion) along with the WT *GFP* sequence, while in the other three, the WT sequence was accompanied by multiple variants. The latter mutations included the maternal 1 bp insertion and, amongst others, a 92 bp deletion plus a 2 bp insertion present in plant #125-SC01 (**Figure 5A**). The direct sequencing of the PCR products derived from the two T7E1-negative regenerants (e.g., #125-SC02) revealed the absence of the WT *GFP* sequence along with a single *GFP* variant in both cases, indicating the likely fixation of the mutant sequence. Overall, 86.7% of the regenerants analyzed were heterozygous or chimeric for a mutation, while the remainders were homozygous. Importantly, both the 1 bp insertion and the 7 bp deletion detected in the T_0_ plant were maintained, and seven additional mutations occurred (**Figure 5A**).

Next, progeny obtained via selfing of the clonal plant #125-SC05 (heterozygous/chimeric) was generated and analyzed to see whether the vegetatively maintained 7 bp deletion can then be sexually transmitted. Genotyping of ten progeny of plant #125-SC05 revealed six plants carrying two or more *GFP* sequences, while the remaining four proved homozygous. Sequencing of these homozygotes as well as two of the heterozygous/chimeric plants uncovered in total three different mutations including the maternal 7 bp deletion (**Figure 5C**).

In addition, the vegetatively generated plants which proved T7E1-negative indicating a homozygous *GFP* mutant allele ought to be confirmed by analyzing their progeny. Toward this end, PCR products of 10 progeny obtained via selfing of plant #125-SC02 were used for direct sequencing and in all of them the maternal 1 bp insertion was recovered in the absence of the *GFP* WT allele (**Figure 5B**). Among these 10 homozygous mutants, three lacked the gRNA/*Cas9*-encoding T-DNA as verified by PCR directed at *Cas9*.

### Variety and Frequency of Mutations

**FIGURE 6:**
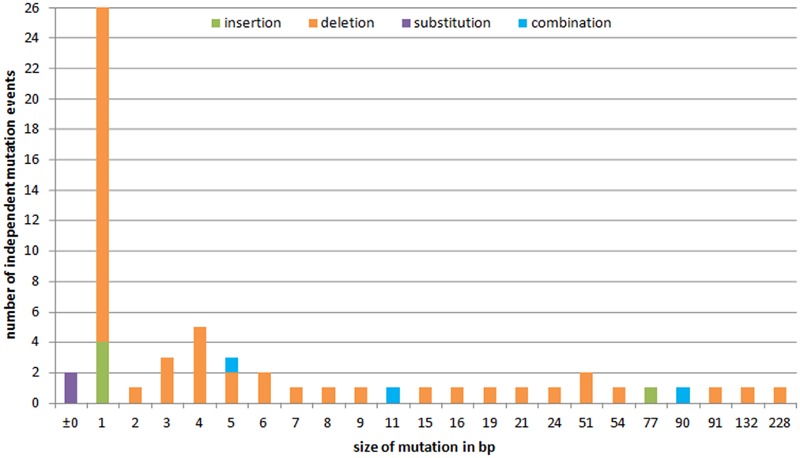
**The variety of induced *GFP* mutations and the frequency of types and lengths of alterations.** The *x* axis plots the extent of the sequence alteration in bp and the *y* axis their frequency. Identical mutations were considered when they derived from independent events.

To investigate the variety of induced mutations and the frequency of independent formation of specific alterations, the data derived from each of the different sets of plant material (T_0_ plants, conventional T_1_ and T_2_ progeny, and regenerants from both, embryogenic pollen culture and leaf explants as well as their progeny) were combined. A total of 59 independent mutation events to the *GFP* sequence was revealed; of these 49 (83.0%) were deletions, 5 (8.5%) were insertions, two (3.4%) were substitutions, and three (5.1%) involved an insertion of 1 or 2 bp in conjunction with a deletion larger than the respective insertion. Three-fourths (77.6%) of the deletions were shorter than 10 bp, and the largest was 228 bp long; except one example (plant #125-14-14, **Figure 3B**), all of the insertions not accompanied by a deletion were of 1 bp in length. Overall, 44.1% of the mutations involved just a single base pair (**Figure 6**).

### Influence of gRNA/Cas9-Induced In-Frame Mutations on the GFP Fluorescence

**FIGURE 7:**
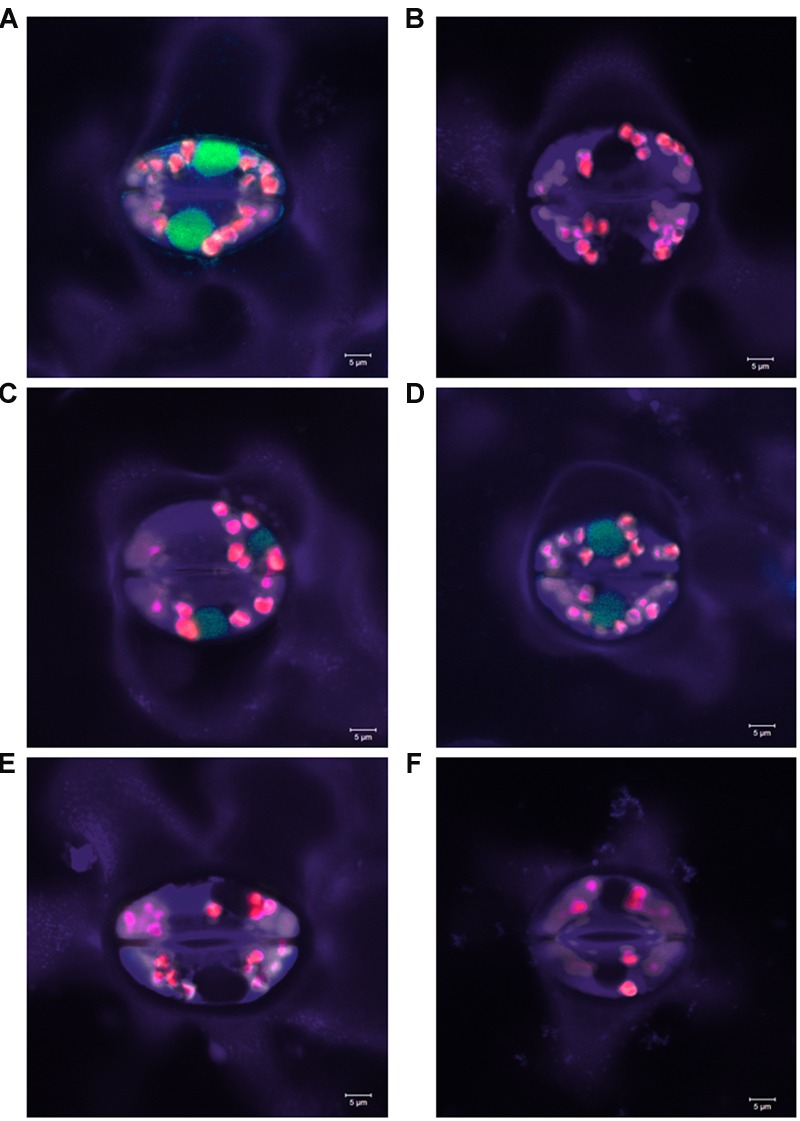
**Green fluorescent protein fluorescence in selected homozygous *GFP* mutants induced by RNA-guided Cas9-endonuclease. (A)** *GFP* WT plant, **(B)** F130G mutant, **(C)** ΔD129 mutant, **(D)** ΔD129/N130V mutant, **(E)** knock-out mutant by frame shift, and **(F)** *Nicotiana tabacum* WT plant. Analysis was performed using a confocal laser scanning microscope (488 nm excitation, 491–530 nm emission).

No reading frame shift was induced in 28.8% of the mutations detected in all the sets of plant material. These mutations included some deletions longer than 2 bp, the 2 bp substitution in plant #125-DH58 and the combined 92 bp deletion/2 bp insertion event in plant #125-SC01. To investigate the effect of in-frame mutations on the GFP fluorescence, selected homozygous mutants were analyzed using confocal laser scanning microscopy. The amino acid sequences of the selected plants are shown in Supplementary Figure S1. Leaf material from a *GFP* WT plant was used as positive control, while a mutant harboring a 1 bp deletion as well as a *N. tabacum* WT plant served as negative controls (**Figures 7A,E,F**). The analysis of the mutants without reading frame shift revealed no residual GFP fluorescence in the F130G (**Figure 7B**) as well as in the Δ18aa/N135D and Δ76aa mutants (pictures not shown). By contrast, GFP fluorescence was still detectable in the mutants ΔD129 and ΔD129/F130V (**Figures 7C,D**), with the intensity being substantially reduced as compared to the *GFP* WT.

## Discussion

Introducing a *GFP*-specific gRNA/*Cas9* construct into tobacco resulted in a high proportion (80%) of the T_0_ plants (those carrying both gRNA and *Cas9* expression units) experiencing one or more mutations to the target locus. A similar level of success has been reported in primary transgenics of the same species, although targeting two different genes (*PDS* and *PDR6*, Gao et al., 2014).

The considerable frequency with which the T-DNA was only partially integrated (in 28.6% of the T_0_ plants) in the present study may reflect the instability of its left border sequence, since the right border sequence is known to be integrated more precisely than the left one (Tinland, 1996; Kim et al., 2003). The vector system used here has also been successful in a number of previous studies; however, no data were given on the proportion of incomplete T-DNA integration (Fauser et al., 2014; Schiml et al., 2014; Wibowo et al., 2016). Whereas placing the selectable marker gene adjacent to the left T-DNA border is thought to result in a high proportion of transgenics carrying the complete T-DNA, the vector used in the present study carried the selectable marker at the right border end of the T-DNA (Fauser et al., 2014). In the few documented cases where the integrity of the *Cas9* and gRNA-containing T-DNA has been investigated, some partial integrations were also observed, and, expectedly, the respective plants proved non-mutant (Zhou et al., 2014; Xu et al., 2015).

Sexual transmission of targeted mutations has not been demonstrated in *Nicotiana* species before. In the present study, the transmission of the mutations to the T_1_ generation mirrors that experienced in both rice (Zhou et al., 2014; Xu et al., 2015) and *A. thaliana* (Feng et al., 2014; Jiang et al., 2014), in that not every mutation revealed in the T_0_ plant (correlates to T_1_ in *A. thaliana*) was re-detectable among the analyzed progeny. For example, the 1 bp insertion present in T_0_ plant #125 was represented in the sample of 50 T_1_ progeny examined, but the 7 bp deletion was not (**Figure 3A**). The 7 bp deletion may have been recovered by an analysis on a larger scale, but due to space and labor capacity limitations, the scope of 50 progeny plants was used in the present study. However, the deletion mutation of plant #125 was maintained both via pollen embryogenesis (**Figure 4A**) and *in vitro* vegetative propagation (**Figure 5A**). Consequently, both of these alternative ways of producing progeny can be considered useful for the recovery of mutations that are rather rarely transmitted to progeny. Beside other Solanaceae species like tomato (McCormick et al., 1986), eggplant (Rotino and Gleddie, 1990) or potato (Chong et al., 1997) also Arabidopsis (Schmidt and Willmitzer, 1988) and apple (James et al., 1989) possess the possibilities for vegetative propagation of gRNA/*Cas9*-induced mutations. Haploid technologies are not restricted to tobacco since doubled haploids can be generated in hundreds of plant species (Kumlehn, 2009). In the context of genome engineering, the employment of haploid technology provides the opportunity to significantly reduce the expenditure of labor, time as well as of laboratory and greenhouse capacities as compared to conventional procedures of plant regeneration and segregation of genetic modifications. In addition to the efficient separation of multiple mutant alleles induced in primary mutants, as was shown in the present study, homozygous mutants can also be directly produced via gene transfer of TALE nuclease to haploid cells, as was previously exemplified by Gurushidze et al. (2014) in barley. The generation of haploid plants does typically not take significantly more time than other *in vitro* plant regeneration methods or the production and germination of seed. Transformations using embryogenic pollen cultures were shown in several species like *N. tabacum* (Sangwan et al., 1993), rapeseed (Fukuoka et al., 1998), and barley (Kumlehn et al., 2006).

Mutations that had not been detected in plant #125 were uncovered among progeny of this plant, irrespective of the mode of propagation. However, it is not known which of these mutations had been present in the T_0_ plant but remained undetected and which ones were newly induced in progeny plants which had inherited the gRNA and *Cas9* expression units along with the *GFP* WT allele. Notably, T_0_ plant #126 appeared to lack any detectable mutations, yet some of its T_1_ progeny harbored mutants, even in the homozygous state (**Figure 3A**). The homozygosity of almost half of these new mutants in the T_1_ generation (**Table 1**) suggests the mutations were likely to have been induced in the T_0_ plant but were not detected at the time of analysis. Note that Zhang et al. (2014) have detected various mutations when DNA prepared from different parts of a T_0_ plant was compared. Two possible scenarios present themselves: first, that the mutations occurred elsewhere in the T_0_ plant or after the DNA had been sampled; and/or secondly, that too small proportion of the T_0_ plant’s DNA analyzed harbored the altered target sequence for the T7E1 assay to pick the effect up. Several false-negative results of the T7E1 assay occurred during the analysis of the progeny. Direct sequencing of the PCR products of these samples uncovered this. Alternatives to the T7E1 assay are, e.g., a PCR/restriction enzyme-based assay or high-resolution melting, but both of these methods are also limited by their relatively low detection sensitivity. By comparison, using deep sequencing, it is possible to detect also rare mutations occurring in small sectors of chimeric plants, provided those sectors are represented in the tissue sample used for the analysis.

The fixation of mutations independent of passage through meiosis cannot, however, be ruled out, given that two of the regenerants from vegetative propagation carried a mutation in the homozygous condition. A possible explanation for this rather unexpected outcome is that a DSB was induced by the RNA-guided Cas9 in the WT *GFP* allele still present in a heterozygous mutant cell, and then was repaired via homologous recombination, recruiting the previously altered sequence as repair template.

Most or perhaps even all of the T_0_ plants were chimeric with respect to mutations at the target site. This result is not surprising, because a shoot regenerating from somatic tissue can originate from more than one cell (Schmülling and Schell, 1993; Li et al., 2009). Moreover, the constitutively expressed RNA-guided *Cas9* in principle can generate a number of independent mutation events in various cells of a developing individual as long as an intact target (WT) allele is present. The resolution of chimeras, as in any mutagenesis program, normally requires passage through meiosis, which can be achieved either via conventional gamete fusion, or, more efficiently, by exploiting the totipotency of immature pollen through the generation of doubled haploids. While ∼60% fixation was achieved by regenerating plants from embryogenic pollen culture, the rate was only half of this among the conventionally generated T_1_ progeny (**Table 1**). Note that no homozygous mutants were obtained among 15 analyzed progeny by the conventional route from plant #254. Similar difficulties have been experienced in fixing mutations induced in *A. thaliana* (Feng et al., 2014; Hyun et al., 2015).

It is worth to mention that the regeneration of plants from embryogenic pollen culture was conducted under Bialaphos-based selection for the gRNA/*Cas9*-harboring T-DNA, since the aim of this investigation was to demonstrate the maximal possible efficiency in the production of homozygous mutants. In contrast to the data presented in **Table 1**, a more conclusive comparison of the proportions of homozygous mutants among doubled haploids generated under selective conditions versus T_1_ derived from selfed T_0_ plants can be made, if only those T_1_ individuals are considered which have inherited the gRNA/*Cas9* T-DNA. The outcome of this simulation of selective conditions for the sexual progeny is as follows; only 31.8% of those T-DNA-positive T_1_ plants proved to carry a mutated target sequence in homozygous conditions, whereas the proportion of homozygous mutants among the doubled haploids was ca. 60%. In this context, it is important to note, that on average two mutations were genetically fixed in T_1_ individuals derived from selfed T_0_ plants, compared to seven fixed mutations as result from the embryogenic pollen culture, which demonstrates that the variety of mutations was much greater in doubled haploids. Given that T-DNA insertions and modified target sequences are typically genetically uncoupled, there is no doubt that homozygous mutants free of T-DNA can be readily produced via the pollen embryogenesis pathway under non-selective conditions. However, the removal of the gRNA/*Cas9*-harboring T-DNA is not required in all applications, since a remaining T-DNA is tolerable, e.g., in gene function studies, provided the target sequence has been mutated and hence is no longer prone to further modifications by the customized endonuclease.

The variety and frequency of induced mutations described here are consistent with the experience of RNA-guided Cas9 applied to rice and tomato, in which a high frequency of small deletions can be induced, along with almost exclusively 1 bp insertions (Zhang et al., 2014; Pan et al., 2016). In *A. thaliana*, it is, however, rather different, since a range in insertion lengths is the outcome (Feng et al., 2014). The basis of this apparent contradiction may lie in species-specific differences in the activity and preferences of the complex endogenous DNA repair mechanisms.

In the present study, no homogeneously biallelic mutant plants were obtained, as was the case in some previous studies targeting genes, e.g., in Arabidopsis (Feng et al., 2013; Jiang et al., 2014), tomato (Pan et al., 2016), and rice (Feng et al., 2013; Zhang et al., 2014). The proportion of biallelic mutations is likely to depend on the overall efficiency of the applied method. Therefore, target sequence and corresponding gRNA are important determinants of mutation efficiency and formation of biallelic mutants. However, some previous studies in rice and Arabidopsis have shown that the occurrence of biallelic mutations is not always correlated with the efficiency of primary mutant formation (Feng et al., 2013; Ma et al., 2015). Rice and tomato are examples where two gRNAs were used per target gene, and in both of these cases only one of the gRNAs was capable of inducing biallelic mutations (Feng et al., 2013; Pan et al., 2016).

Furthermore, biallelic and homozygous mutants should preferentially be identified or confirmed after generative transmission, because unambiguous evidence of a biallelic mutation can be provided only via segregation analysis. This was demonstrated in previous studies where primary transgenic plants, in which only one or two mutant alleles and no WT had been detected, nonetheless produced progeny carrying further mutant alleles and/or the WT allele (Zhang et al., 2014; Xu et al., 2015). In addition, Xu et al. (2015) found that one of the two mutant alleles of such tentatively biallelic plants was not transmitted to the progeny, which strongly indicated a preliminarily undetected chimeric nature of the primary mutant. Whereas mutation events taking place in leaf primordia can be well represented in sampled leaf tissue, the core of the shoot apical meristem, which gives rise to the gametophytes (germline cells), may remain only partially or even non-mutated. Consequently, a major limitation in the characterization of primary mutants is that whatever sequencing method is used, it cannot be non-destructively applied to the entire plant. And, on the other hand, whatever sample is used for sequencing the target, it is not necessarily representative for the whole, possibly chimeric mutant. A more detailed look at publications in which biallelic mutations were reported reveals that sometimes only few individually subcloned PCR products were sequenced, while no progeny analysis was conducted to rule out chimerism (Brooks et al., 2014; Ma et al., 2015).

A great proportion of the induced *GFP* mutations did not lead to a reading frame shift. The analysis of the GFP fluorescence of these mutants showed that the deletion of 18–96 amino acids led to the loss of the GFP signals, while the deletion of amino acid D129 as well as the mutation ΔD129/F130V caused a reduction of the fluorescence (**Figure 7**). According to Flores-Ramírez et al. (2007), the amino acid D129 is part of the longest loop of the GFP protein (I128-L141) and most of the deletions in this region destroyed GFP fluorescence due to inappropriate folding of the protein. In the same work, these authors also showed that the mutant ΔD129 conserved the GFP signal, albeit at a lower level than the WT, which was caused by a reduced amount of protein. This could also be an explanation for the reduced fluorescence in the plants analyzed in the present study. In the plant harboring the 2 bp substitution which caused the F130G exchange, no GFP signal was detectable. Likewise, in the study of Flores-Ramírez et al. (2007), it was shown that the exchange of phenylalanine on position 130 is tolerated only with hydrophobic residues. Since in the present study F130 was replaced by glycine, the loss of GFP fluorescence could be explained by reduced protein stability, due to the fact that F130 is important for the fixation of the longest loop of the GFP protein.

In summary we have demonstrated that gRNA/Cas9-induced mutations in *N. tabacum* can be efficiently transmitted to progeny and fixed using haploid technology, that mutations found in primary transgenic plants can be maintained and rendered heritable via somatic tissue culture and that in-frame mutations can be used to attenuate protein function, which extends the options site-directed mutagenesis offers in the context of functional validation of genes of interest as well as for the improvement of crop performance.

## Author Contributions

JK conceived the study. SS, SP, GH, AM, and TR generated the necessary plasmids, performed the experiments, and analyzed the data. SS and JK wrote the manuscript. All authors amended the manuscript.

## Conflict of Interest Statement

The authors declare that the research was conducted in the absence of any commercial or financial relationships that could be construed as a potential conflict of interest.
