# Supplementary material for: RNA-Guided Cas9-Induced Mutagenesis in Tobacco Followed by Efficient Genetic Fixation in Doubled Haploid Plants
Source: Front Plant Sci. 2017 Jan 4;7:1995. doi: 10.3389/fpls.2016.01995 (PMC5209389; doi:10.3389/fpls.2016.01995)
Supplement: Supplementary file 1 [file Table_1.docx]

Supplementary Material

RNA-guided Cas9-induced mutagenesis in tobacco followed by efficient genetic fixation in doubled haploid plants

**Sindy Schedel, Stefanie Pencs, Götz Hensel, Andrea Müller, Twan Rutten and Jochen Kumlehn^*^**

*** Correspondence:** Dr. Jochen Kumlehn: kumlehn@ipk-gatersleben.de

**Table S1:** Primers used for the identification of T-DNA regions, the amplification of the *GFP* target region and the cloning of binary vectors pGH292 and pSI24.

| **Primer** | **Sequence 5’-3’** | **Amplified region** |
| --- | --- | --- |
| Cas9 F2 | CAGCTCGTGCAGACCTACAAC | *Cas9* gene – forward primer |
| Cas9 R2 | TGCCTTCTAAGGATAGCGTG | *Cas9* gene – reverse primer |
|  |  |  |
| AtU6-26 F1 | CAGCTAGAGTCGAAGTAGTG | gRNA – forward primer |
| 35S P R1 | GACAGATAGCTGGGCAATGG | gRNA – reverse primer |
|  |  |  |
| 35S P F1 | GACGTAAGGGATGACGCAC | *BAR* gene – forward primer |
| BAR R2 | GAGACGTACACGGTCGACTC | *BAR* gene – reverse primer |
|  |  |  |
| GH-GFP R2 | TACGGCAAGCTGACCCTGAA | *GFP* gene – forward primer |
| GH-GFP F1 | GGTCACGAACTCCAGCAGGA | *GFP* gene – reverse primer |
|  |  |  |
| GH-SpeI-GFP F1 | TAACTAGTATGGTGAGCAAGGGCGAGGAGCTG | *GFP* gene – forward primer |
| GH-NcoI-GFP R1 | TACCATGGAATACTTGTACAGCTCGTCCATGCCG | *GFP* gene – reverse primer |
|  |  |  |
| GFP proto Fw | ATTGGCTGAAGGGCATCGACTTCA | protospacer sequence forward |
| GFP proto Rev | AAACTGAAGTCGATGCCCTTCAGC | protospacer sequence reverse |
