# Supplementary material for: RNA-Guided Cas9-Induced Mutagenesis in Tobacco Followed by Efficient Genetic Fixation in Doubled Haploid Plants
Source: Front Plant Sci. 2017 Jan 4;7:1995. doi: 10.3389/fpls.2016.01995 (PMC5209389; doi:10.3389/fpls.2016.01995)
Supplement: Supplementary file 2 [file Image_1.pdf]

```
GFP WT        CGCATCGAGCTGAAGGGCATCGACTTCAAGGAGGACGGCAACATCCTG
                R  I  E  L  K  G  I  D  F  K  E  D  G  N  I  L
               122 123 124 125 126 127 128 129 130 131 132 133 134 135 136 137

125-DH58-01   CGCATCGAGCTGAAGGGCATCGACGGCAAGGAGGACGGCAACATCCTG
                R  I  E  L  K  G  I  D  G  K  E  D  G  N  I  L   F130G

125-14-01     CGCATCGAGCTGAAGGGCAT---CTTCAAGGAGGACGGCAACATCCTG
                R  I  E  L  K  G  I  -  F  K  E  D  G  N  I  L   ΔD129

126-40-06     CGCATCGAGCTGAAGGGCATCG---TCAAGGAGGACGGCAACATCCTG
                R  I  E  L  K  G  I  -  V  K  E  D  G  N  I  L   ΔD129/F130V

254-08-12     ----------------------------------------ACATCCTG
                -  -  -  -  -  -  -  -  -  -  -  -  -  D  I  L   Δ18aa/N135D

125-DH61-05   ------------------------------------------------
                -  -  -  -  -  -  -  -  -  -  -  -  -  -  -  -   Δ76aa

126-05-04     CGCATCGAGCTGAAGGGCATCGAC-TCAAGGAGGACGGCAACATCCTG
                R  I  E  L  K  G  I  D   S  R  R  T  A  T  S     KO mutant
```

Figure S1: Comparison of the GFP amino acid sequences in selected homozygous *GFP* mutants induced by RNA-guided Cas9. The sequence colored blue is the protospacer sequence and the one colored green is the protospacer-adjacent motif (PAM). Deletions are represented by red dashes and insertions by red letters. The amino acids are consecutively numbered as indicated in the third line. Amino acid changes and/or deletions are indicated to the right of each sequence. The last sequence shown is of a knock-out mutant carrying a 1 bp deletion which causes a frame shift and the formation of premature stop codon.
